# Protected Stroke Mechanical Thrombectomy Code During the Coronavirus (COVID-19) Pandemic: Southwestern Part of Saudi Arabia Stroke Unit Local Protocol

**DOI:** 10.7759/cureus.7808

**Published:** 2020-04-24

**Authors:** Saeed A Alqahtani, Ibrahim Alnaami, Adel Alhazzani

**Affiliations:** 1 Neurology, College of Medicine, King Khalid University, Abha, SAU; 2 Neurosurgery, College of Medicine, King Khalid University, Abha, SAU; 3 Medicine, College of Medicine, King Saud University, Riyadh, SAU

**Keywords:** covid 19, stroke systems of care, re-vascularization, coronavirus pandemic, tretment protocol

## Abstract

Cerebrovascular diseases are a significant cause of mortality and morbidity worldwide, in particular those with large vessels occlusion (LVO). Coronavirus disease 2019 (COVID-19) has become a global crisis rapidly since its initial outbreak in Wuhan, China, in December, 2019. Stroke due to LVO needs rapid assessment and timely endovascular intervention which can be very challenging during the time of pandemic where you need to deliver proper, safe, and timely care to acute ischemic stroke (AIS) patients with LVO, yet, protecting healthcare workers and existing patients at the medical facility. In this article, we share our local experience in the stroke unit at Aseer Central Hospital which is the main hub of stroke patients in the southwestern part of Saudi Arabia and the primary regional COVID center to provide guidance to perform smooth, safe, and swift mechanical thrombectomy during the coronavirus (COVID-19) pandemic as well as possible similar future situations.

## Introduction

The pandemic of the coronavirus disease (COVID-19) has significant negative consequences on clinical practice worldwide. One of the significant challenges was to deliver proper, safe, and timely care to acute ischemic stroke patients (AIS) with large vessel occlusion (LVO), yet, protecting healthcare workers and existing patients at the medical facility [1].

Following the start of the COVID-19 pandemic, the Saudi Patient Safety Center (SPSC) established priority codes for medical illnesses and operative procedures in collaboration with scientific societies across the Kingdom of Saudi Arabia [2]. Healthcare stakeholders in SPSC have established four priorities codes. Code red includes patients with acute ischemic stroke and they have to be managed urgently and swiftly. The stroke unit at Aseer Central Hospital is the main hub of stroke patients in the southwestern part of Saudi Arabia. Herein, we established along with local healthcare authorities a Protected Stroke Mechanical Thrombectomy (PSMT) code in which the management and the service of stroke patients remain unaffected, ensure the safety of healthcare workers and patients without compromising the capacity of the vacant beds for anticipated new COVID-19 patients.

## Technical report

Methods

Aseer Central Hospital is the primary stroke hub in the southwestern part of Saudi Arabia. The hospital is providing tertiary medical care for an estimated population of two million. The hospital became a comprehensive stroke unit in February 2019. The stroke unit operated by a multidisciplinary team from different neurosciences background. Due to the global coronavirus (COVID-19) disease pandemic and to lessen potential infectious hazards to the medical teams, stroke physicians, neuro-interventional team and other patients, a group of vascular neurology, neurosurgery, emergency room physicians, anesthesia, neuro-interventional physicians, along with local healthcare taskforce were gathered to develop a modified protected stroke code for stroke patients with large vessel occlusion. All members were board-certified in their specialties. The encounters were conducted through teleconferencing platforms. Each group of the team was asked to develop a modified algorithm to the pre-existing local stroke mechanical thrombectomy code acknowledging the new COVID-19 disease pandemic. The Protected Stroke Mechanical Thrombectomy (PSMT) code aims to facilitate the timely necessitated acute stroke intervention and protecting the healthcare providers. The modified protocol was agreed upon by each group based on the best medical shared knowledge, clinical practice, available literature review, and the final consensus among the team. The final approval to launch the protected stroke mechanical thrombectomy code was obtained from the local healthcare taskforce.

Results

The PSMT code in our local medical institution was efficiently executed without significant obstacles. The current protocol was helpful as guidance to perform smooth, safe, and swift mechanical thrombectomy during the COVID-19 pandemic as well as possible similar future situations.

## Discussion

Stroke due to LVO needs a rapid assessment and timely endovascular intervention [1]. Strokes with LVO remain a top neurological emergency even during a medical crisis because of the high mortality rate and severe neurological disability. The global pandemic crisis of the highly contagious COVID-19 outlines a unique challenge to any medical organization. Since the number of confirmed COVID-19 patients is globally increasing, neurologists, and especially neuro-interventionalists who attend the stroke codes, should pay attention to the safety protocols. Here we present our local experience in dealing with acute stroke patients with LVO who need urgent mechanical thrombectomy (MT) during the pandemic of COVID-19. We are a new comprehensive stroke center established by the Ministry of Health (MOH) in February 2019, with a total of 50 cases of mechanical thrombectomies till the end of March 2020. Also, our hospital is the only ready COVID-19 center in Aseer region (southwestern part of Saudi Arabia). This paper will focus only on acute stroke patients who need urgent mechanical thrombectomy. We established a protected stroke mechanical thrombectomy (PSMT) code (Table 1) in order to organize all the efforts and ensure the safety of all involved medical personnel dealing with stroke patients who need MT.

**Table 1:** Protected Stroke Mechanical Thrombectomy (PSMT) Framework.

***Protected Stroke Mechanical Thrombectomy Code (PSMT)***
* Assume all stroke patients with large vessel occlusion (LVO) are COVID-19 infected patients until proven otherwise
* All medical staff approaching the stroke patients should use Personal Protection Equipment (PPE): (eye protection, face shield, surgical gown, gloves, shoe covers, and a fit-tested N95 mask)
* Place surgical mask on the patient's mouth (make sure you place your own PPE first)
* Perform focused neurological examination and limit the examinations that may compromise the contact or airborne precautions
* All stroke patient with LVO are prophylactically intubated in the emergency room following a safe intubation protocol before transportation to angiography suite or computed tomography (CT) scan room
* Inside the dedicated COVID-19 angiography suite: Only one neuro interventionalist, one nurse and one interventional radiology technician are allowed and wearing all standard PPE
* Outside dedicated COVID-19 angiography suite: Anesthesia staff member wearing standard PPE, Emergency room nurse for charting wearing standard PPE
* After the stroke mechanical thrombectomy is over, the patient has to be transferred back to the emergency room (recovery room)
* No extubation is allowed in the angiography suite
* Patient to be transferred outside our COVID-19 center as soon as possible to a designated local hospital with ICU admission
* Planned safe protocol extubation is preferable once stroke patient arrived in the outside hospital local ICU for better neurological evaluation
* All stroke mechanical thrombectomy patients are monitored and co-managed with the outside local ICU team using Tele-means

Furthermore, due to the exceptionally increasing number of COVID-19 patients, and unlikely to have COVID-19 test status before the mechanical thrombectomy, we assumed that every stroke patient with LVO who presented to our stroke center is having COVID-19 until proven otherwise. Our hospital is applying a strict COVID-19 visual and clinical screening to all visitors and patients. However, we use extra safety measures, particularly toward stroke patients with LVO due to the inherited complex clinical presentation, which includes but not limited to the decreased level of consciousness, aphagia, and sometimes difficulties in obtaining collateral needed clinical information.

Older patients with prior cerebrovascular disease, stroke risk factors, and cardiovascular disorders are at higher risk to contract COVID-19 infection [2]. There is evidence that COVID-19 can cause neurological symptoms, and approximately five percent of severely affected COVID-19 patients may develop acute ischemic stroke [3,4].

Since our center is COVID-19 ready center and with the expected rise in the confirmed positive cases globally, we accommodated a transitional protected stroke mechanical thrombectomy protocol to minimize the strain on our health system, proper intensive care unit (ICU) utilization, and to maximize care to the patients.

Protected stroke mechanical thrombectomy (PSMT)

All eligible stroke patients with LVO are transferred to our stroke center to receive only the acute mechanical thrombectomy. After the stroke mechanical thrombectomy, the patients will be transferred to a local hospital with ICU admission to maximizing the availability of ICU beds for COVID-19 patients when needed. We continued to provide stroke care by adopting Tele-means. We approach all stroke patients presented to our center who need MT as COVID-19 suspected cases until proven otherwise. In such scenarios, we use recommended standard personal protective equipment (PPE) precautions (eye protection, face shield, surgical gown, gloves, shoe covers, and a fit-tested N95 mask) before approaching any stroke patients [5,6]. To protect healthcare workers, these precautions are applied to any staff members directly dealing with stroke patients (neurologists, neuro-interventionalists, emergency room staff, angiography suite nurses, and technicians). We perform focused neurological examination and try to limit the neurological examinations that may compromise the contact or airborne precautions such as repetitive language assessment or cerebellum examination [7-9]. We try to minimize the numbers of the medical team to the minimum, and the primary neuro-interventionalist on board is the one in charge as a safety leader.

To lessen the patient's movement/agitation, airborne respiratory droplets, or vomiting, which all carry high-risk exposure to the medical team, we prophylactically intubated stroke patients with LVO, especially those with a decreased level of consciousness (Glasgow Coma Scale less than 10), high NIHSS, posterior circulation stroke, or dominant left-sided cerebral hemisphere stroke before mobilizing patients to the angiography suite [10]. We adjudicated to hasten the MT procedural time with a goal of safe, swift, and quick MT and not to spend more than 30 minutes from groin puncture to full and successful recanalization, therefore, alternating schedule among two board-certified neuro-interventionalists. One is the primary on-call 24/7 for one week, and the other is working as a backup for emergencies or in a case of accidental exposure to COVID-19 high-risk patients in order to minimize the risk of infections.

After finishing the stroke MT procedure, patients will be taken back to the emergency room (recovery room). Then the stroke patients will be transferred as soon as possible to a different local hospital with ICU admission. All patients will not be allowed to be extubated in the angiography suite. Upon patients' arrival to the outside hospital ICU, and when appropriate, the planned safe protocol extubation is succeeded in order to obtain a better neurological assessment [11-13].

In our COVID-19 center, only one first degree relative is allowed to be with the patient, and sometimes family is not available or unreachable, and given that stroke endovascular MT is a timely procedure, in this instance, we waived the family consent for the MT procedure. Instead, we obtain two different attending consultant signatures.

We have two angiography suites; we have dedicated one biplane angiography suite as "COVID-19 Suite". All non-urgent neuroendovascular procedures are canceled. The dedicated suite is stockpiled with all required PPE and stroke endovascular interventional materials to minimize procedural interruption. The angiography suite is adequately sanitized after each case.

## Conclusions

The crisis caused by the COVID-19 disease pandemic has resulted in a significant strain on the medical system. Providing timely acute intervention for stroke patients with large vessel occlusion during the COVID-19 pandemic is very challenging, considering the potential spread of infection among the medical teamwork. Protected stroke mechanical thrombectomy (PSMT) code in our local hospital was efficiently executed without significant obstacles. The current protocol is helpful as guidance to perform smooth, safe, and swift mechanical thrombectomy during the COVID-19 pandemic as well as possible similar future situations. The protocol is a safety and assurance framework to prevent accidental or unknowingly exposure and yet optimizing medical care.
